# Antiplatelet Therapy for Stent-Assisted Coil of Ruptured Middle Cerebral Artery Bifurcation Aneurysm: Is There a Right Answer?

**DOI:** 10.7759/cureus.11612

**Published:** 2020-11-21

**Authors:** Megan M Finneran, Michael Young, Hamad Farhat

**Affiliations:** 1 Neurological Surgery, Carle BroMenn Medical Center, Normal, USA; 2 Neurological Surgery, Advocate Health Care, Oak Lawn, USA; 3 Neurological Surgery, Advocate Christ Medical Center, Oak Lawn, USA

**Keywords:** anticoagulation, mca bifurcation aneurysm, stent-assisted coil

## Abstract

A variety of modalities exist for treatment of cerebral aneurysms. Stent-assisted coiling is an effective option but poses a challenge regarding antiplatelet therapy. No consensus exists among neuroendovascular surgeons regarding preferred agent, dose, and timing to balance the risk of thromboembolism and hemorrhage. This is especially true in the setting of aneurysmal subarachnoid hemorrhage.

We present a 66-year-old female with history of thrombocytopenia and nonalcoholic cirrhosis who presented with severe headache. Head CT demonstrated a right temporal lobe intraparenchymal hemorrhage with sylvian fissure subarachnoid hemorrhage. Cerebral angiogram showed a 1.5mm x 1.5mm right middle cerebral artery (MCA) bifurcation aneurysm. The patient underwent Y-stent coiling from the right M1 into the right M2 superior division and the right M1 into the right M2 inferior division, with a 1mm x 1cm coil. Given the patient’s thrombocytopenia, only aspirin monotherapy was administered peri-procedural. Shortly thereafter, the patient developed left hemiparesis. Computed tomography angiogram (CTA) demonstrated thrombus within the stent. Thrombectomy was performed with thrombolysis in cerebral infarction (TICI) 3 revascularization and improvement to neurologic baseline. However, that evening she became acutely hypotensive, unresponsive, and ultimately expired due to hemorrhagic cause.

Antiaggregate therapy among neuroendovascular procedures is debated with no clear standard of care. This case highlights the difficult decisions that must be made to balance the risks associated with the use of antiplatelets with ruptured aneurysms.

## Introduction

Treatment and outcomes of aneurysmal subarachnoid hemorrhage (aSAH) have changed drastically since the advent of endovascular techniques [1]. Stent-assisted coiling is a commonly used modality, but is not without risks [2]. Antiplatelet therapy is used to mitigate the potential complication of thromboembolic event [3]. Its use is particularly controversial in the setting of aSAH due to the risk of bleeding complications [4]. Despite these known consequences, there is no standard for agent, dose, or timing to optimize the risk of thromboembolism and subsequent hemorrhage. We present a unique case in which the advantages and disadvantages of various antiplatelet therapies occurred in the same patient in the span of hours.

## Case presentation

A 66-year-old female presented to the emergency department complaining of severe right frontotemporal headache, left facial numbness, and nausea. Earlier that day she developed a headache and began dropping objects. Medical history included non-alcoholic liver cirrhosis and thrombocytopenia with baseline platelet count of 60,000-80,000/mcL.

On physical examination the patient was alert, oriented, and in no apparent distress. She presented as Hunt Hess grade 2 with mild facial droop and mild dysarthria. Her strength was intact in both upper and lower extremities. She had left hemi-sensory loss. 

**Figure 1:**
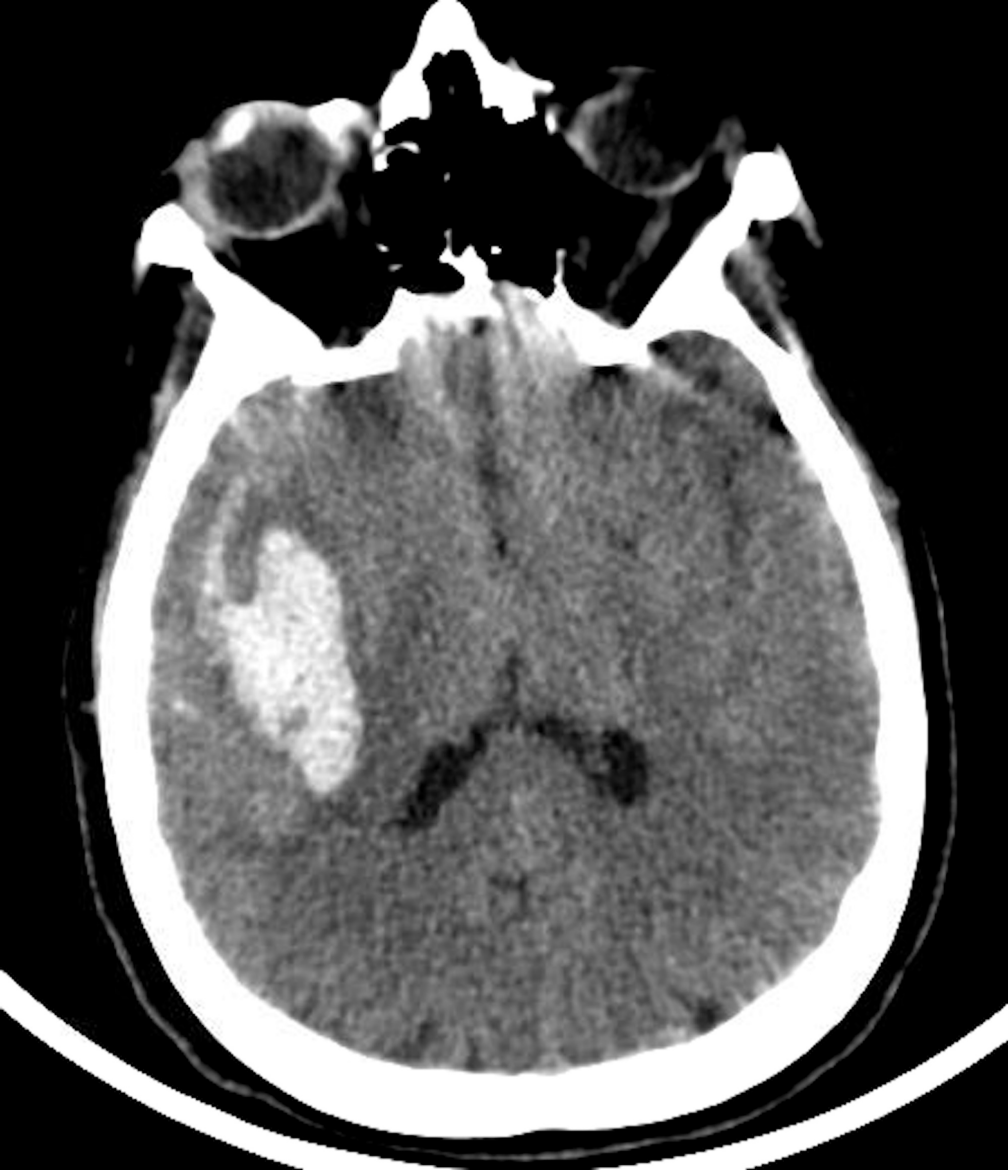
Initial non-contrast head computed tomography (CT) demonstrates right temporal lobe intracerebral hemorrhage with subarachnoid hemorrhage within the sylvian fissure.

Head computed tomography (CT) without contrast demonstrated a 4.5cm x 2.9cm x 2.2cm right temporal intraparenchymal hemorrhage with subarachnoid hemorrhage into the sylvian fissure (Figure 1). Computed tomography angiogram (CTA) was negative for aneurysm.

Platelet count was 69,000/mcL, hemoglobin was 12.6g/dL, and international normalized ratio (INR) was 1.3. Aspartate transaminase (AST) was 79 IU/L and alanine transaminase (ALT) 40 IU/L. The patient received two units of platelets; post-transfusion platelet count was 67,000/mcL.

**Figure 2:**
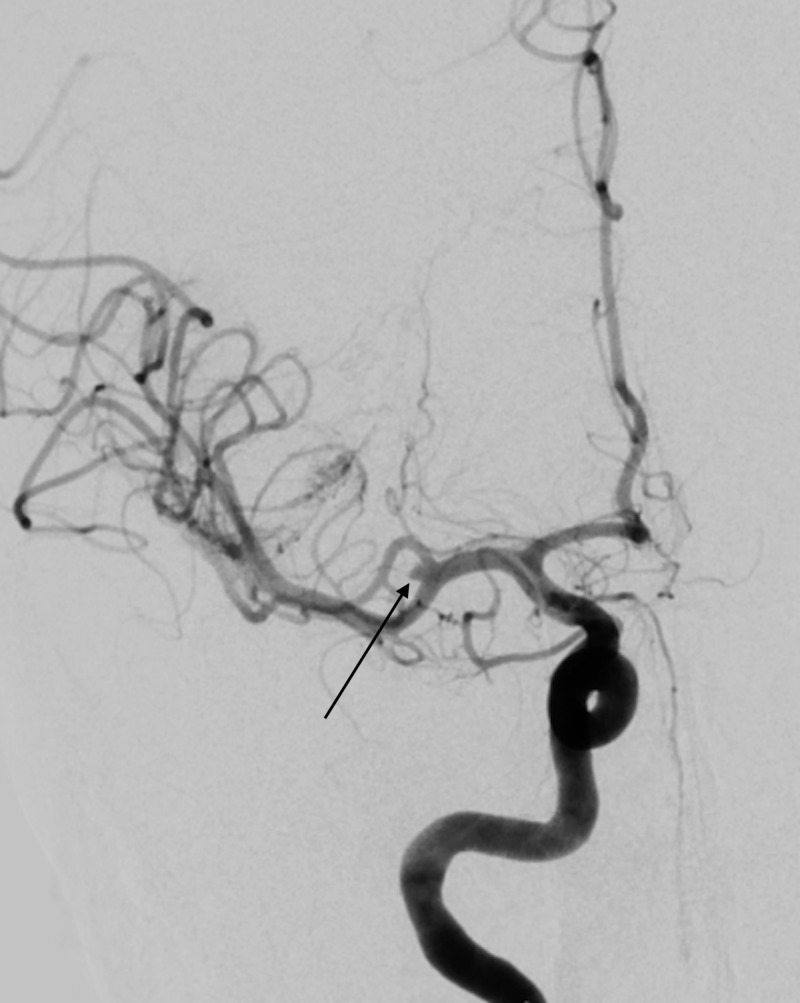
Anterior posterior digital subtraction angiogram (DSA) shows a 1.5mm x 1.5mm blister right middle cerebral artery bifurcation aneurysm.

Digital subtraction angiogram (DSA) demonstrated a 1.5mm x 1.5mm right middle cerebral artery (MCA) bifurcation aneurysm (Figure 2). Vascular access was obtained through the right femoral artery and a 6 French Angioseal (Terumo, Somerset, NJ, USA) was used to close. No groin hematoma occurred. The following day, the patient was loaded with aspirin 325mg. She received two units of platelets; post-transfusion count was 135,000/mcL.

**Figure 3:**
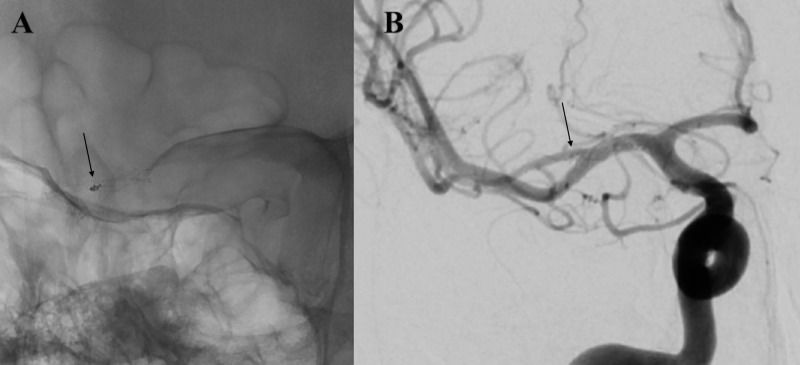
A, left: Unsubtracted anterior posterior angiogram shows the Y-stent construct and coil placed within the aneurysm. B, right: Anterior posterior angiogram post-stent coiling of blister right middle cerebral artery aneurysm identifies no further filling of the aneurysm and patency of the M1 and M2 divisions of the right middle cerebral artery.

The next morning the patient was taken to the angiogram suite, intubated, and put under general anesthesia. The right common femoral artery was cannulated and an 8 French vascular sheath was inserted. A 6 French x 80cm neuron max (Penumbra, Alameda, CA, USA) was placed within the vascular sheath. The vascular sheath, guide sheath, and diagnostic catheter were perfused with heparinized saline solution throughout the procedure. A 5 French Berenstein catheter (Cordis, Miami Lakes, FL, USA) with a 0.038-inch glidewire (Terumo) was advanced under fluoroscopy into the right common carotid artery. An SL-10 microcatheter (Stryker, Kalamazoo, MI, USA) was advanced over a Synchro 2 microwire (Stryker) into the right internal carotid artery (ICA). Using roadmap guidance, the right MCA bifurcation artery aneurysm was selectively catheterized. A Headway 17 microcatheter (Microvention, Aliso Viejo, CA, USA) was then prepared and advanced over a Synchro 2 microwire. At that point, using roadmap guidance, a 2.5mm x 13mm low-profile visualized intraluminal support (LVIS) Junior stent (Microvention) was deployed from the right M1 to inferior M2 division. Another Headway 17 microcatheter (Microvention) was prepared and advanced over a Synchro 2 microwire (Stryker) and, using roadmap guidance, a 2.5mm x 17mm LVIS Junior stent (Microvention) was deployed from the right M1 to superior M2 division. A single 1mm x 1cm Penumbra SMART coil was then advanced through the jailed SL-10 (Stryker) microcatheter. A final angiogram showed no filling of the aneurysm and patency of both stents (Figure 3); an 8 French Angioseal (Terumo) was used. The patient awoke with persistent mild left facial droop and dysarthria but no other neurological deficits. With the patient’s thrombocytopenia and cirrhosis, additional antiplatelet therapy was held.

Within 30 minutes of extubation, the patient developed new left hemiparesis and right gaze preference. CTA showed complete occlusion of the right distal M1 to the proximal superior division of the M2 within the previous Y-stent construct. She was immediately taken to the angiography suite.

**Figure 4:**
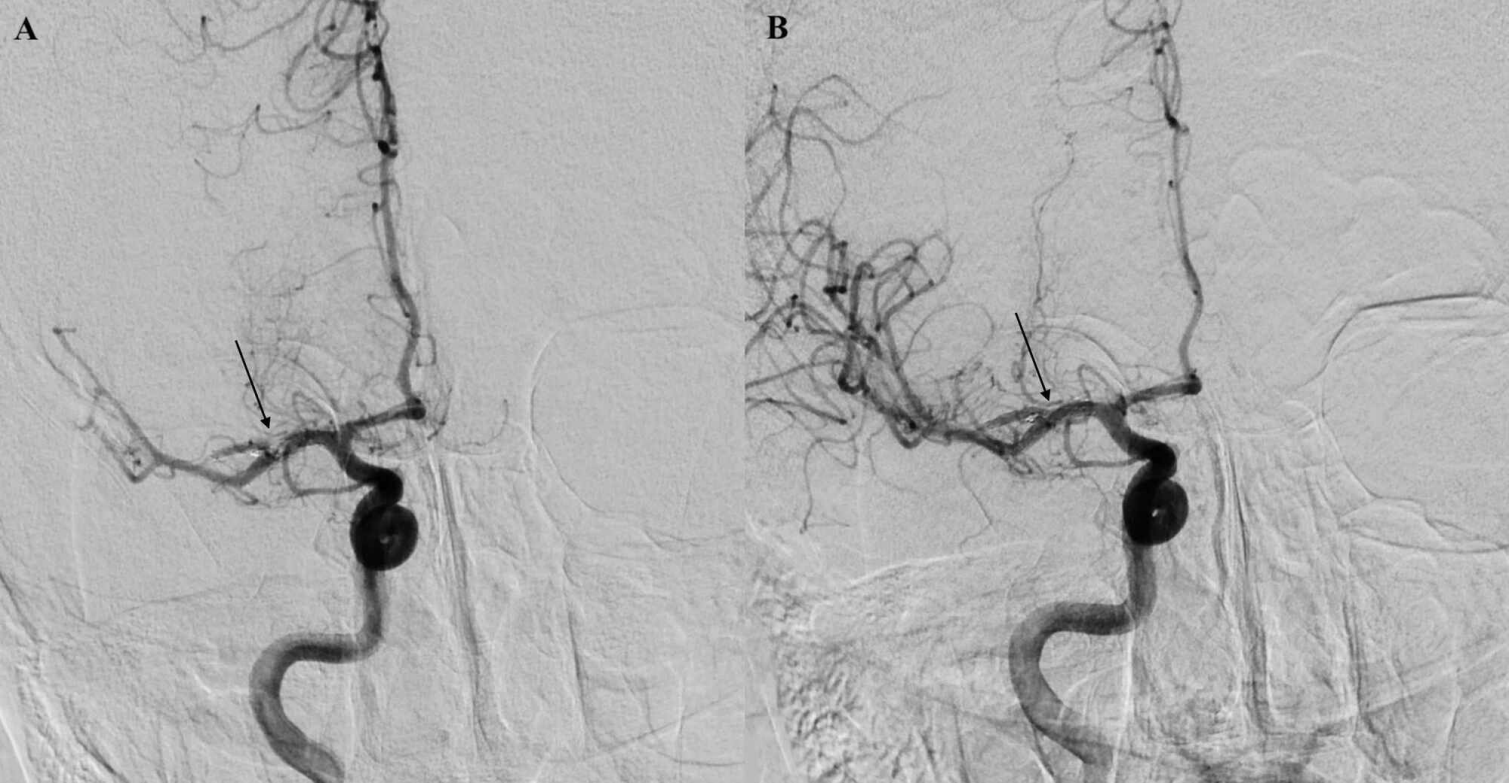
A, left: Anterior posterior angiogram identifies a thrombus within the stent in the M1 to superior M2 division of the right middle cerebral artery. B, right: Anterior posterior angiogram post-aspiration thrombectomy reveals revascularization of the right middle cerebral artery.

The right common femoral artery was cannulated. An 8 French vascular sheath and a 6 French Neuron Max sheath (Penumbra) were placed. A 5 French angled Berenstein (Cordis) catheter with a 0.038 inch Terumo Glidewire was advanced under fluoroscopy into the right common carotid artery and subsequently the right ICA. The thrombus within the Y-stent construct was visualized, causing the superior M2 occlusion (Figure 4A). Next, a 5 French Sofia aspiration catheter (Microvention) was advanced over a Synchro 2 (Stryker) microwire against the thrombus. Aspiration was performed with thrombolysis in cerebral infarction (TICI) 3 revascularization (Figure 4B). An 8 French Angioseal (Terumo) was used to close.

Immediately after the procedure, the patient’s left hemiparesis had improved; she was anti-gravity in the left upper extremity and lower extremities. A slight facial droop persisted. Post-procedure hemoglobin was 10.7g/dL; platelet count was 58,000/mcL. She received one dose of clopidogrel 75mg and an eptifibatide bolus with weight-based infusion dosed for four hours. Daily aspirin 325mg and clopidogrel 75mg were planned to start the following day. 

Approximately three hours after completion of the thrombectomy, the patient became acutely hypotensive with poor response to norepinephrine. The groin dressing was saturated with a palpable hematoma. The eptifibatide was stopped and a massive transfusion protocol was initiated. The patient received multiple units of platelets and packed red blood cells, in addition to sodium bicarbonate and albumin. Despite maximum therapy, the patient remained persistently hypotensive. Family ultimately elected for palliative extubation and the patient expired. 

## Discussion

The cause of death for this patient was never determined; the presumed differential included an intracerebral hemorrhage (ICH), a retroperitoneal hemorrhage, or a cardiac event. Given the patient’s persistent hypotension despite transfusion, a hemorrhagic event appeared more likely. This case prompts a discussion about antiplatelet therapy in the setting of intracerebral hemorrhage, aneurysmal subarachnoid hemorrhage, thrombocytopenia, and the need for intracranial stenting.

Hemorrhage was our greatest initial concern. Ruptured aneurysm patients are at higher risk for hemorrhagic complications compared to unruptured aneurysm patients [3]. The patient’s presentation of aSAH with associated intraparenchymal hemorrhage, combined with her medical history of thrombocytopenia and cirrhosis further complicated the decision regarding antiplatelet therapy. These confounding factors increased her risk of hemorrhagic complication, beyond that of simply a ruptured aneurysm. In an attempt to decrease this risk, we elected to load with single antiplatelet therapy (aspirin) the day before the procedure and planned to withhold further antiplatelet therapy until the following day. In consulting the literature, this approach is a reasonable one.

One study that assessed post-procedural duration for antiplatelet therapy found two of 43 patients developed mild hemiparesis within less than 24 hours after stent-assisted coil embolization. Both patients were diagnosed with cerebral infarction in the area of the stented vessel, despite receiving dual antiplatelet therapy (DAPT) [5]. The study did not comment on hemorrhagic complications. In regards to our patient, this study demonstrated the potential for early thromboembolic events despite DAPT.

Our attempt to prevent hemorrhage led to another well-established complication of neuroendovascular surgery: thromboembolism. It is impossible to know if early DAPT could have prevented this event. In a study that encompassed a survey of comprehensive stroke centers, the majority of centers used aspirin and clopidogrel DAPT as standard of care. Alternatively, four institutions used monotherapy: two used aspirin, one used clopidogrel, and one used a single agent, unspecified [6]. Endovascular procedure-specific details were not included in the surveys. However, it demonstrated that there is no clear consensus among neuroendovascular surgeons regarding optimal therapy.

The risk of hemorrhage and overall complications is higher among ruptured aneurysms than non-ruptured. One review of aSAH patients with stent-assisted coiling who received DAPT found ICHs in 8% of patients, one-third of which were external ventricular drain (EVD)-associated hemorrhages. They found clinically significant thromboembolic events in 6% of patients [7]. The overall procedure-related complication rate was ≤13%, which included hemorrhagic and thromboembolic complications. Alternatively, another study reported a complication rate of 7% in stent-assisted coiling of unruptured aneurysms [8]. 

Upon identification of the thrombus and subsequent thrombectomy, we began clopidogrel and eptifibatide to prevent further thromboembolic events. In the context of published reports, this treatment modality was also appropriate and may have been considered initially.

One study among aSAH patients treated with coiling compared intraprocedural 650mg aspirin followed by 14 days of daily aspirin 325mg with no aspirin or other form of antiplatelet or anticoagulation beyond intraprocedural intravenous heparin. Enrollment in the control group was terminated early due to the increased rate of thromboembolic events in the first 72 hours after treatment [3]. Zero major bleeding complications were cited in either group, but they did find a higher incidence of minor GI bleeding and EVD track hemorrhage. Stent-assisted coiling patients were excluded from the study, which preclude us from directly applying the study to our patient. Still, the study does demonstrate an increase in thromboembolic events in the absence of antiplatelet treatment in patients who undergo endovascular treatment. 

In evaluating acutely ruptured aneurysms that underwent endovascular treatment without antiplatelet premedication, a stent-assisted coiling group was compared with coiling without stent assistance. Procedural thromboembolism occurred more frequently in the stent-assisted coiling group but treatment outcomes were comparable [9]. All patients in both groups received DAPT with aspirin and clopidogrel immediately after embolization for a minimum of three months. This study shows the use of stent-assisted coiling compared with coiling alone further potentiates the risk of thromboembolism.

In the end, it seemed neither choice led to a favorable outcome for our patient. Without antiplatelet therapy, she developed a thromboembolic event. With it, she developed a probable hemorrhagic complication that resulted in death. We reflect on this case with no clear answer as to how best to serve patients like this one.

## Conclusions

A range of treatment options exist for intracranial ruptured aneurysms. Stent-assisted coiling is an effective method, albeit one that is not without risks. Antiplatelet therapy is frequently used, but details regarding optimal agent, dose, and timing are unclear in the literature. This case of a 66-year-old woman with a small ruptured MCA bifurcation aneurysm treated with stent-assisted coiling suffered from two complications: a thromboembolic event and presumably a hemorrhage that resulted in mortality. Further research and communication among the endovascular community is needed to identify the optimal way to treat patients with antiplatelet therapy while minimizing harm. 
